# Capillaroscopy as an Outcome Measure for Clinical Trials on the Peripheral Vasculopathy in SSc—Is It Useful?

**DOI:** 10.1155/2010/784947

**Published:** 2010-08-16

**Authors:** Maurizio Cutolo, Alberto Sulli, Carmen Pizzorni, Vanessa Smith

**Affiliations:** ^1^Research Laboratory and Academic Unit of Clinical Rheumatology, Department of Internal Medicine, University of Genova, Viale Benedetto XV, 6, 16132 Genova, Italy; ^2^Department of Rheumatology, Ghent University Hospital, 9000 Ghent, Belgium

## Abstract

Peripheral microvascular impairment in systemic sclerosis (SSc) may be easily detected and scored in a safe noninvasive way by nailfold videocapillaroscopy (NVC). The paper highlights clinical conditions related to SSc in which NVC may represent an outcome measure of therapeutical interventions, by elaborating on their already assessed relationship with the NVC patterns and eventually scores. The 3 important biological/clinical conditions are: the positivity for SSc-specific serum autoantibodies, the presence of SSc skin digital ulcers (DUs) and of pulmonary arterial hypertension (PAH) SSc associated. In conclusion, to the question if capillaroscopy (NVC) may represent in SSc an outcome measure for clinical trials on the peripheral vasculopathy, based on the growing evidence and our detailed studies, the answer is positive. Recent therapeutic trials in SSc are confirming this role, and the experience is growing rapidly.

## 1. Introduction

**Figure 1:**
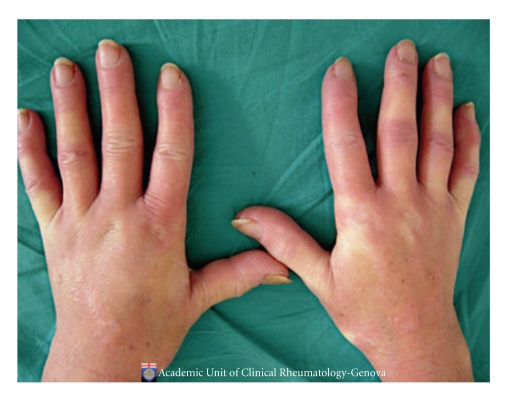
Hands of a patient with early systemic sclerosis suffering from secondary Raynaud's phenomenon.

Systemic sclerosis (SSc) is characterized by early and persistent microvascular impairment leading to functional Raynaud's phenomenon (RP) and clinical manifestations (i.e., digital ulcers, pulmonary arterial hypertension, etc.) (Figure 1) [1, 2]. 

Digital ulcers in SSc are considered to be related to tissue ischemia following several processes, including at the beginning persistent vasospasm (RP), but in the progression of the disease also to intimal fibroproliferation, tissue fibrosis, and thrombosis of digital arteries [3]. 

Progressive deficiency in vasodilatory capacity of the vessels and tissue fibrosis is proposed as a mechanism of the persistent vascular spasm; however, the mechanism of endothelial injury is still unclear [4]. 

**Figure 2:**
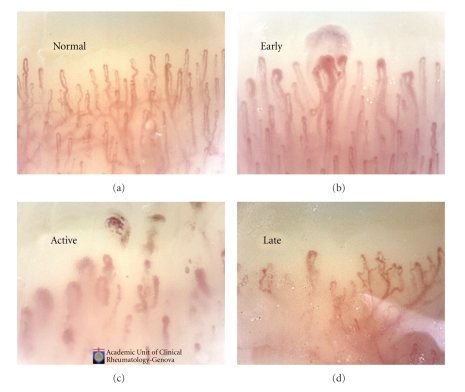
The morphological capillary abnormalities in SSc have been classified in 3 validated patterns (early, active, and late) of microangiopathy by NVC analysis.

The assessment of vascular involvement is still a matter of study, and several noninvasive techniques have been proposed. Peripheral microvascular impairment in SSc may be easily and safely detected by nailfold videocapillaroscopy (NVC). The morphological capillary abnormalities in SSc have been classified in 3 validated patterns (early, active, and late) of microangiopathy by NVC and scored (Figure 2) [5–7]. 

NVC may partially observe the column of red blood cells moving inside the capillary, but the technique does not allow measurement of the blood flow. 

Laser Doppler flowmetry (LDF) is the best non invasive and safe technique to assess and to measure the blood perfusion at peripheral sites [8, 9]. 

Blood flow has been found to be reduced in patients with SSc, compared with healthy subjects and patients with primary RP. Patients with SSc showing the late NVC pattern of microangiopathy have a significantly lower finger blood perfusion (FBP) than patients with the active and early NVC patterns (*P* < .05) [10]. 

The question today is if capillaroscopy (and eventually LDF) may represent an outcome measure for clinical trials on the peripheral vasculopathy in SSc. 

We will analyze clinical conditions related to SSc in which NVC may represent an outcome measure by considering their already assessed relationship with the NVC patterns and/or eventually scores. The 3 important biological/clinical conditions are: the SSc-specific serum autoantibodies, the SSc skin digital ulcers (DUs), and the pulmonary arterial hypertension (PAH) associated to SSc.

## 2. Serum Autoantibodies and NVC

SSc is characterized by serum autoantibodies, including anticentromere (anti-CENP-B), anti-Th/To, antitopoisomerase I (anti-topo I), and anti-RNA polymerase I/III (anti RNAP III). Together, these markers account for almost 85% of autoantibodies specific for SSc and show a predictive value for clinical evaluation and prognosis [11, 12]. 

Anti-CENP-B and anti-topo I are known predictors of progression from isolated RP to SSc [13]. However, until recently, many of the studies on the significance of expression of these antibodies in SSc have been limited by small sample sizes, incorrect classification of patients with manifestations of connective tissue disorders as having primary RP, use of varying definitions of subsets of patients, lack of standardised methods for determining antinuclear antibodies, omission of tests for anti-Th/To and anti-RNAP III antibodies, and absence of multivariable analyses. 

Antiendothelial cell antibodies (AECAs) are a heterogeneous class of antibodies whose role in the pathogenesis of autoimmune diseases with vascular involvement has been extensively studied and are present in the serum samples of many patients with SSc (22–86%) but are not SSc specific [14]. Even if, among the demonstrated clinical associations, lung and peripheral vascular involvement is the most common, further research on this topic, including longitudinal studies in patients with SSc, is mandatory for a better understanding of the clinical value of AECA. 

However, for long time it has not been determined prospectively whether SSc autoantibodies are related to the course and type of microvascular damage detectable by nailfold capillaroscopy. 

LeRoy and Medsger proposed that patients with RP who had abnormal findings on NVC and SSc-specific autoantibody should be classified as having early SSc [15]. This set of criteria had not been validated and considered for long time, until recently. 

Finally, Koenig et al. prospectively studied a large cohort of Raynaud's patients who were referred to a single centre for evaluation of RP over a period of 20 years [16]. 

 The objectives were to identify the strongest independent predictors of progression to definite SSc, to determine the type and time course of microvascular damage by nailfold capillaroscopy and its relationship to major SSc autoantibodies, and to validate the criteria for early SSc. 

Of the 586 patients who were followed up for 3,197 person-years, 74 (12.6%) developed definite SSc. 

In fact, this study validated the criteria of LeRoy by demonstrating that almost all patients who were to futurely develop SSc had “early” SSc (Raynaud's phenomenon plus a scleroderma pattern on capillaroscopy and/or SSc-specific antibodies) at the baseline visit.

Concerning the scleroderma pattern they reported a characteristic sequence of microvascular damage, starting with enlarged capillaries (giant capillaries) that identify the “early” SSc pattern, followed by capillary loss that indicates the “active” SSc pattern and then by capillary telangiectasias (neoangiogenesis) that might better characterize the “late” SSc pattern (Figure 2), from the stadium of “early” SSc until development of definite SSc. 

Definite SSc was diagnosed in close temporal relationship to capillary loss. 

Enlarged capillaries (giant capillaries), capillary loss, and SSc-specific autoantibodies independently predicted definite SSc. 

Interestingly, anti-CENP-B and anti-Th/To antibodies predicted for the development of giant capillaries; these autoantibodies and anti RNAP III also predicted for capillary loss. Each autoantibody was associated with a distinct time course of microvascular damage.

At followup, 79.5% of patients with one of these autoantibodies and abnormal findings at the baseline nailfold capillaroscopy examination had developed definite SSc. Patients with both baseline predictors were 60 times more likely to develop definite SSc. 

These data validated the proposed criteria for early SSc. In conclusion, in the presence of a secondary RP evolving to definite SSc, microvascular damage (as assessed by nailfold capillaroscopy) is dynamic and progressive, and SSc-specific autoantibodies are associated with the course and type of capillary abnormalities. 

It was confirmed that the microvascular damage in secondary RP evolving to definite SSc is characteristically sequential, starting with enlarged capillaries (giant capillaries, “early” SSc pattern) followed by capillary loss (“active” SSc pattern), and then by capillary telangiectasias (neoangiogenesis, “late” SSc pattern). 

Since new therapeutic agents are being evaluated in patients with SSc, awareness of this sequence of microvascular damage has potential implications for future trials. Of note, recently, it was established the evolution of microangiopathy in “established” SSc disease, by pointing out the progression of capillary loss and augmentation of ramifications in a microangiopathy score [7]. 

The final message is that abnormal findings on nailfold capillaroscopy at baseline together with a systemic sclerosis-specific autoantibody indicate a very high probability of developing definite systemic sclerosis whereas their absence excludes this outcome [17]. In SSc patients awareness of this sequence of microvascular damage and SS-associated autoantibodies has potential implications for future therapeutical trials. 

For example, in trials of novel angiogenic, vasculogenic, or fibrosis-modulating agents, it would appear realistic to select SSc patients at a uniform stage of microvascular damage and with similar SSc-specific autoantibodies and eventually to evaluate such damage longitudinally by NVC to assess response to treatment.

## 3. Skin Digital Ulcers and NVC

**Figure 3:**
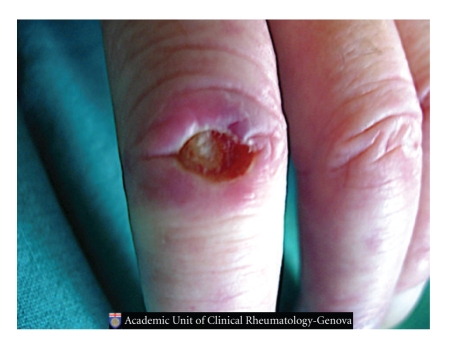
Classical skin digital ulcer in a patient affected by systemic sclerosis.

Skin DUs represent one of the most frequent clinical manifestations of microangiopathy in patients with SSc (Figure 3) [18]. 

On the other hand, a decreased number of capillary loops should be considered highly specific for advanced RP, and it has been estimated that the number of normal capillaries may be reduced to just 20% in patients with active SSc. Skin DUs seem to be associated with the “late” NVC pattern, characterized by avascular areas (severe capillary loss). 

Recently, using a semiquantitative score that highlights the importance of the number of capillaries, an association was reported between advanced stages of capillary loss (mean score class 2 and 3) and digital trophic lesions in 49% of patients with SSc [19]. Also, loss of capillaries may be relevant in determining tissue hypoxia, and, in patients with recent onset of RP, the appearance of rapidly progressive capillary loss may represent the first capillaroscopic evidence of severe SSc with destruction of microvessels [20]. 

The extensive disappearance of capillaries may generate large avascular areas giving a “desert-like” appearance to the nailfold bed, and progressive loss of capillaries has been associated with more extensive skin involvement (as well as diffuse SSc) and a poor prognosis.

 As a consequence, the early detection of SSc patients who are at high risk of developing DU could allow preventive treatment of these complications with reduction of morbidity and social costs. 

Very recently, it was found, in 130 SSc patients examined at entry and after 20 months of follow-up, that the diffuse cutaneous form of SSc with avascular areas on capillaroscopy represented, among other factors (e.g., increased interleukin- 6) the major risk factor for DU development [21]. 

A previous study showed that, patients with late SSc pattern at NVC showed an increased risk to have an active disease (odds ratio (OR) 3.50; 95% confidence interval (CI) 1.31–9.39) and to present skin DU (OR 5.74; 95% CI 2.08–15.89) [2]. 

Another recent investigation showed that a quantitative capillaroscopic score was suggested highly predictive of the development of new skin DU within 3 months after NVC [22]. The predictive value of this index still needs to be confirmed in a validation study. 

A clinical history of multiple skin DU is the most helpful predictor and indicator for preventive therapy, and the loss of capillary as assessed at NVC has been found the best possible NVC predictive marker to be considered. 

However, the routine use of NVC now seems a possible predictive tool to enable the early detection of patients at a high risk of developing skin DU [1]. In this regard, at present, the cost/effectiveness ratio for the therapy of SSc skin DU is very unfavourable, and strategies for their treatment or prevention are under debate [2].

## 4. Pulmonary Arterial Hypertension and NVC

SSc is the main connective tissue disease associated with PAH and PAH is estimated to affect 12% of SSc patients, being the leading cause of death in this disease [23]. 

Structural changes in the systemic microcirculation, consisting of a reduction of capillary density and widening of the capillaries, are considered an hallmark of SSc. 

It is now clear that the severity of this microvascular damage as assessed by NVC differs between patients with SSc and PAH (SSc-PAH) and those with SSc without PAH (SSc-non-PAH) and correlates with pulmonary haemodynamic parameters. 

Interestingly, when compared to healthy controls, the same is true for patients with idiopathic PAH, a condition not known to be characterized by systemic microvascular changes [24]. 

A recent study suggests that capillary density reduction is a marker of the presence and severity of PAH [24]. 

A few studies have investigated nailfold capillary patterns in patients with SSc-PAH, with only one study including patients with idiopathic PAH. Two studies used echocardiography and/or right heart catheterisation to confirm the diagnosis of PAH. One of these, by Ong et al., found a significant reduction of capillary density in eight patients with SSc-PAH in comparison with 12 patients with SSc-non-PAH [25]. Pulmonary haemodynamic parameters were not reported in the study by Ong. 

The other study, by Greidinger et al. using capillary density and qualitative scoring of nailfold patterns, found no differences in capillary patterns between eight patients with SSc-non-PAH and seven with SSc-PAH, but capillary density in these groups was not reported [26]. 

A third study, by Ohtsuka et al. using only right heart catheterisation to diagnose and exclude the diagnosis of PAH, showed a significant difference in semiquantitative scoring of nailfold patterns between SSc-non-PAH and SSc–PAH patients, but, again, capillary density was not assessed in this study [27]. 

Practically, only one of these studies included patients with idiopathic PAH and reported no differences in capillary density and capillary patterns between 13 healthy controls and 37 patients with idiopathic PAH. 

There is, however, more recent evidence of a reduction of capillary density in both SSc-PAH and, albeit to a milder extent, in idiopathic PAH [24]. 

The explanation for a reduction in capillary density may not be the same for the two disorders. In SSc it is generally presumed that structural changes in the systemic (micro)circulation precede changes in the pulmonary circulation, as systemic microvascular changes may precede the development of SSc by many years. 

Therefore, NVC abnormalities might also reflect what is going on in the pulmonary circulation. This may not be true for all capillary abnormalities, because most patients with SSc demonstrate nailfold capillary abnormalities whereas only a minority develop PAH. Recent data confirm that only capillary density is associated with the presence of PAH and is a marker of disease severity in SSc [24]. 

A further suggested explanation for the more pronounced capillary reduction in SSc-PAH could be that PAH itself amplifies the reduction of capillary density already present in SSc. 

However, Houben et al. recently observed an increase rather than a decrease of nailfold capillary density in patients with heart failure [28]. 

The observation that circulating plasma levels of endothelin1 (ET-1) are raised in patients with PAH and that ET-1 production is increased in the pulmonary tissue of affected individuals makes this vasoconstrictor a particularly interesting target for therapeutic intervention in PAH [29]. 

Clinical trials with ET receptor antagonists have clearly shown that such antagonists provide symptomatic benefit in patients with PAH, thereby proving the clinical relevance of the endothelin system as a therapeutic target with optimised use of selective ETA or nonselective ETA/ETB blockade. 

As matter of fact the highest serum ET-1 levels are found in SSc with late NVC pattern and visceral involvement [30]. 

Recent therapeutical examples of clinical trials with NVC as measure of outcome and conclusions are presented. 

Very recent studies represent examples of clinical trials in which NVC has been successfully included as a measure of treatment outcome. 

The objective of one study was to evaluate NVC pattern changes in SSc patients treated regularly on cyclic basis with iloprost and to find associations with clinical, serologic, and pharmacological variables [31]. 

Forty-nine patients affected by SSc underwent two NCV analyses at 3 years apart from each other. Six patients showed an amelioration of NVC abnormalities who changed from active to early pattern; five of these cases (83.3%) had been given cyclophosphamide therapy and the remaining case methotrexate plus azathioprine. 

Cyclophosphamide administration was significantly associated with regression of the NVC pattern (*P* < .001). Interestingly, none of the SSc patients who received cyclophosphamide demonstrated worsening of the microvascular lesions; the progression of NVC pattern was inversely correlated to cyclophosphamide treatment (*P* = .02). 

Therefore, cyclophosphamide treatment demonstrated to be effective in modulating the SSc microvascular damage as directly observed and monitorized by NVC. 

In another study cyclophosphamide treatment showed to be effective for SSc microvascular damage as directly observed by rapid improvement of the NVC pattern [32]. 

Confirmation of the trend was obtained by a further study on autologous stem cell transplantation that confirmed a significant regression of the NVC pattern together with the improvement of the clinical conditions [33]. 

In conclusion, to the question if capillaroscopy (NVC) may represent in SSc an outcome measure for clinical trials on the peripheral vasculopathy, based on the growing evidences and our detailed studies, the answer is positive. 

Early recent therapeutic trials in SSc are confirming this role, and the experience is growing rapidly [34].

To definitely establish its role as an outcome measure two requirements still need to be fulfilled. First, large multicentric and longitudinal and randomized controlled trials (certainly in establishing its role as an outcome measure in therapeutical trials) are needed. Second, multicentric reliability in scoring systems is warranted. 

Recently, a first step in assessing reliability has been taken through demonstration of reliability both in qualitative and semiquantitative assessment of nailfold images of SSc patients in a bicentre setting [5].
